# Virus-host interaction networks as new antiviral drug targets for IAV and SARS-CoV-2

**DOI:** 10.1080/22221751.2022.2071175

**Published:** 2022-05-23

**Authors:** Na Chen, Baoge Zhang, Lulu Deng, Bing Liang, Jihui Ping

**Affiliations:** a MOE Joint International Research Laboratory of Animal Health and Food Safety, Engineering Laboratory of Animal Immunity of Jiangsu Province, College of Veterinary Medicine, Nanjing Agricultural University, Nanjing, People’s Republic of China; b College of Veterinary Medicine, Nanjing Agricultural University, Nanjing, People’s Republic of China

**Keywords:** IAV, SARS-CoV-2, virus-host interactions, replication cycle, host innate immunity, antiviral drug targets, Omicron

## Abstract

Currently, SARS-CoV-2, especially the Omicron strain, is ravaging the world and even co-infecting human beings with IAV, which is a serious threat to human public health. As of yet, no specific antiviral drug has been discovered for SARS-CoV-2. This requires deeper understandings of the molecular mechanisms of SARS-CoV-2-host interaction, to explore antiviral drug targets and provide theoretical basis for developing anti-SARS-CoV-2 drugs. This article discussed IAV, which has been comprehensively studied and is expected to provide the most important reference value for the SARS-CoV-2 study apart from members of the Coronaviridae family. We wish to establish a theoretical system for the studies on virus-host interaction. Previous studies have shown that host PRRs recognize RNAs of IAV or SARS-CoV-2 and then activate innate immune signaling pathways to induce the expression of host restriction factors, such as ISGs, to ultimately inhibit viral replication. Meanwhile, viruses have also evolved various regulatory mechanisms to antagonize host innate immunity at transcriptional, translational, post-translational modification, and epigenetic levels. Besides, viruses can hijack supportive host factors for their replication. Notably, the race between host antiviral innate immunity and viral antagonism of host innate immunity forms virus-host interaction networks. Additionally, the viral replication cycle is co-regulated by proteins, ncRNAs, sugars, lipids, hormones, and inorganic salts. Given this, we updated the mappings of antiviral drug targets based on virus-host interaction networks and proposed an innovative idea that virus-host interaction networks as new antiviral drug targets for IAV and SARS-CoV-2 from the perspectives of viral immunology and systems biology.

## Introduction

The COVID-19 pandemic has spread rapidly throughout the world since its outbreak, leading to 400 million infections and about 6 million deaths worldwide. Aside from its severe damage to the global economy and public health, SARS-CoV-2, the virus responsible for the COVID-19, could even co-infect human beings with IAV. Recently, Omicron, a variant of SARS-CoV-2, has become a global focus. It was discovered in early November 2021 and quickly spread worldwide, becoming the main epidemic strain in some countries and causing social panic. Additionally, the influenza virus, another virus that seriously affects human health, should not be underestimated. As a common respiratory pathogen, it causes seasonal epidemics and severe occasional worldwide pandemics, thereby resulting in human casualties and significant economic losses. It is recorded that four influenza pandemics have occurred over the past century [1]. The 1918 Spanish H1N1 influenza pandemic infected about 1 billion people and caused more than 50 million deaths worldwide, the most serious harm to humanity. The other influenza pandemics occurred in 1957 (Asian influenza, H2N2), 1968 (Hong Kong influenza, H3N2), and 2009 (H1N1) [1]. Influenza viruses are enveloped RNA viruses and belong to the family Orthomyxoviridae. There are four types of influenza viruses: A, B, C, and D [2]. Among all subtypes, IAV is the most common, whose genomes are composed of eight negative-sense single-stranded RNA segments. Six segments among the eight encode their corresponding proteins: HA, NA, NP, PA, PB1 and PB2, while the remaining two segments can encode two corresponding proteins by variable splicing, namely M1, M2, and NS1, NEP, respectively [3]. All the 10 proteins mentioned above are essential for IAV replication. In addition, non-essential proteins are also encoded by IAV, such as PA-X, and PB1-F2 (i.e. dispensable for IAV replication, but rather modulate host immune response) [4]. The genome of IAV is an ssRNA, and the dsRNA is produced during viral replication. Moreover, the vRNPs consist of eight vRNAs, NP and RNA polymerase complexes composed of PA, PB1, and PB2. In conclusion, SARS-CoV-2 and IAV have a serious impact on human public health. Therefore, developing antiviral drugs and the updating of antiviral drug targets remain an urgent and long-term need.

The discovery of TLRs, the first kind of pattern recognition receptors identified in the innate immune signaling pathway, was awarded the 2011 Nobel Prize in Physiology or Medicine, which has fuelled the interest in gaining a deeper knowledge of innate immunity, especially the race between host antiviral innate immunity and viral antagonism of host innate immunity. After almost a decade of in-depth exploration, biomolecular interaction networks consist of various host biomolecules associated with IAV replication (such as PRRs, adaptor proteins, signaling proteins, transcription factors, interferons and their receptors, JAK-STAT-SOCS, host restriction factors, supportive host factors, microRNAs, lncRNAs, circRNAs, vtRNAs, sugars, lipids, hormones, and inorganic salts) have been initially formed [2,5–9]. The mechanisms of innate immune responses against IAV have been studied, while the numerous strategies of IAV to antagonize innate immunity have also been revealed [10]. Furthermore, with the support of transcriptomics, proteomics, metabolomics, epigenetics, and single-cell sequencing, a comprehensive analysis of biomolecular regulatory networks associated with IAV replication was conducted at the chromatin, DNA, transcriptional, translational, and post-translational modification levels [7,10]. Thus, a relatively complete theoretical system of virus-host interaction networks has gradually been formed. Notably, current studies on the IAV-host interaction network involve multiple disciplines, such as viral immunology, molecular biology of viruses, system biology, biochemistry, physiology, network pharmacology, bioinformatics, and structural biology. In conclusion, fundamental components of virus-host interaction networks include the virus, host, and viral antagonism of host innate immunity. Among them, viral proteins are traditional antiviral drug targets.

The potential antiviral drug targets are important for developing effective antiviral drugs. Most approved antiviral drugs target a viral enzyme that plays an essential role in viral replication. In addition, host cell pathways and virus-host interactions are being used as antiviral targets [11–13]. To date, only two classes of antiviral drugs are globally approved and available for the treatment of influenza infections: M2 ion channel blockers and NA inhibitors. The first class includes adamantane derivatives, amantadine, and rimantadine, which inhibit proton conductivity of the M2 ion channel of IAV, hence preventing the viral uncoating step of the viral replication cycle. However, they are often associated with limited efficacy and adverse side effects. In addition, the currently available drugs suffer from rapid and extensive emergence of drug resistance. All this highlights the urgent need for developing new antiviral strategies with novel mechanisms of action and with reduced drug resistance potential [14,15]. Viruses rely on host cellular functions to replicate, and therefore a thorough understanding of the roles of virus-host interaction networks during IAV replication is essential to develop new anti-IAV drugs [16]. Unlike the development strategies of new anti-IAV drugs based on virus-host interaction networks proposed by some scholars [16–18]. Initially, the host targets summarized in this review include non-coding RNAs, sugars, lipids, hormones, and inorganic salts, rather than being limited to proteins. What's more, we systematically summarized IAV antagonism of host innate immunity, which is an often-overlooked antiviral drug target. Finally, we elucidated the molecular mechanisms of IAV-host interaction from the perspectives of viral immunology and systems biology rather than bioinformatics. As of yet, no specific antiviral drug has been discovered for SARS-CoV-2. Therefore, scholars have discussed the potential antiviral drug targets for SARS-CoV-2 extensively, which include viral targets (especially spike protein), host targets, and virus-host protein interactions [19–21]. However, host targets are mainly limited to host receptors that mediate viral entry, while ignoring biomolecules such as host factors, glycans, lipids, and inorganic salts that regulate SARS-CoV-2 replication. In addition, the viral antagonism of innate immune signaling pathways is easy to be neglected. As components of interaction networks, these overlooked potential antiviral drug targets have also been discussed in this review. What's more, the current studies on the SARS-CoV-2-host interaction network are still in its initial stage. Considering that IAV has the advantages of a relatively systematic theoretical system and has many similarities with SARS-CoV-2. Important inspirations initiated from the research results of IAV-host interaction network are summarized to accelerate the study's progress on SAR-CoV-2-host interaction network and lay a theoretical foundation to develop anti-SARS-CoV-2 drugs.

**Figure 1.**
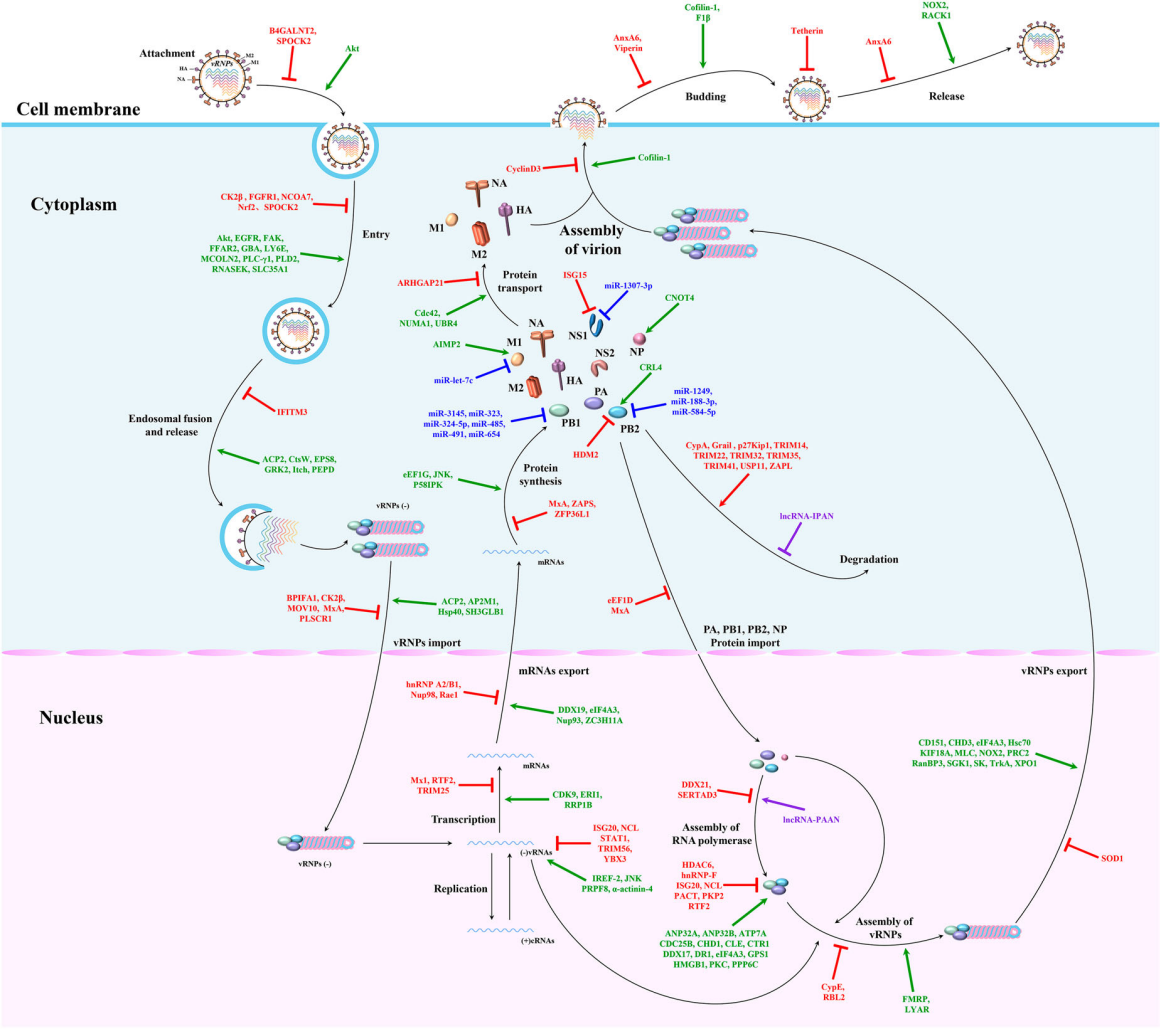
The mapping of antiviral drug targets focusing on IAV replication cycle. A complete IAV replication cycle consists of four stages: attachment, entry and uncoating, replication and transcription, assembly and release. Host restriction factors, supportive host factors, microRNAs, and lncRNAs can regulate IAV replication by directly acting on one or multiple steps of the IAV replication cycle. Theoretically, these host biomolecules, which regulate the viral replication cycle and each of these steps in the viral replication cycle, could be used as potential antiviral drug targets. Green lines indicate that supportive host factors target one or multiple steps of the IAV replication cycle. Red lines indicate that host restriction factors target one or multiple steps of the IAV replication cycle. Purple lines indicate that lncRNAs target one step of the IAV replication cycle. Blue lines indicate that microRNAs target IAV proteins.

**Figure 2.**
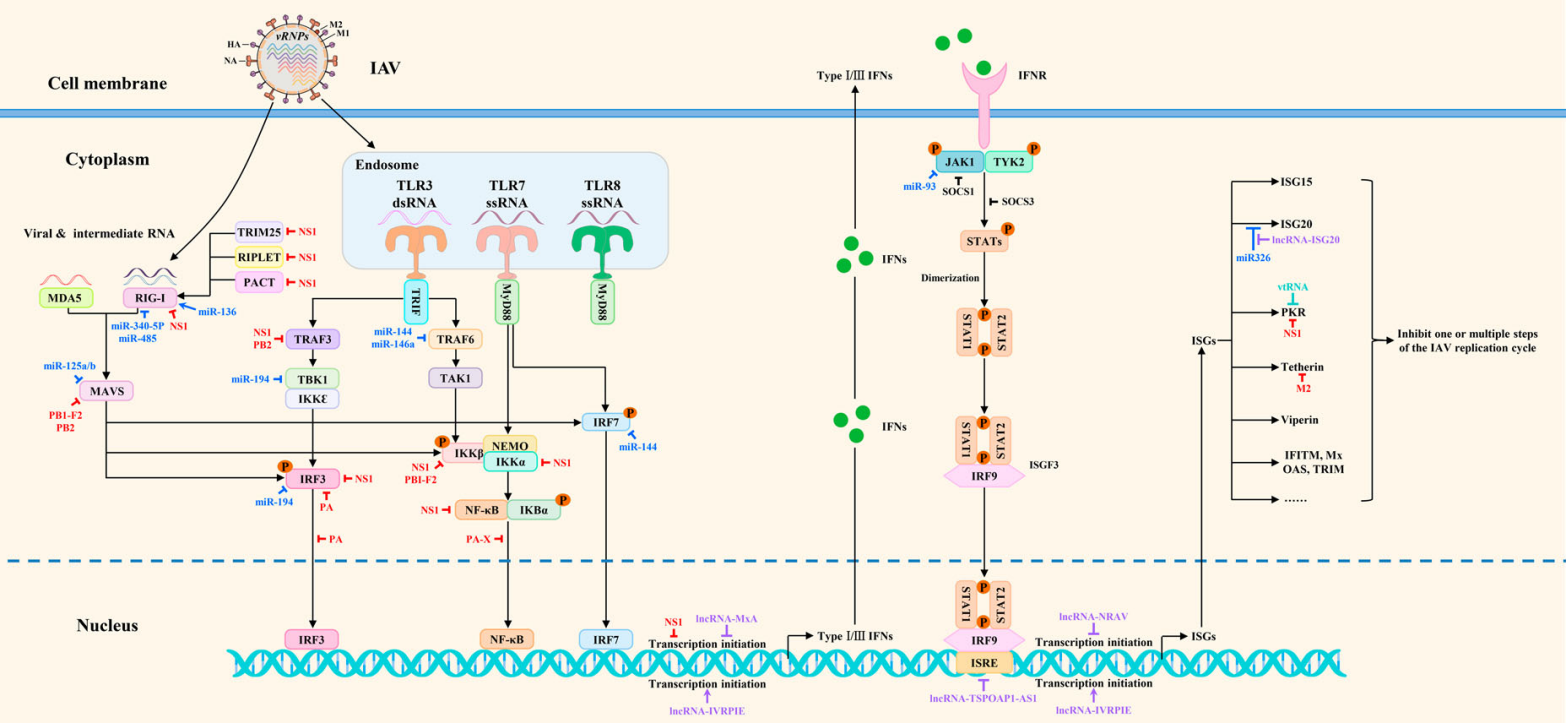
The mapping of antiviral drug targets focusing on host innate immune responses against IAV. The host PRRs can recognize dsRNA or ssRNA during IAV replication and quickly activate the innate immune signaling pathways to induce the expression of interferons and downstream ISGs, thereby inhibiting one or multiple steps of the IAV replication cycle. Meanwhile, IAV has evolved multiple strategies to directly or indirectly antagonize host innate immunity at transcriptional, translational, post-translational modification, and epigenetic levels. Notably, microRNAs, lncRNAs, and vtRNAs regulate innate immune signaling pathways. Theoretically, these host biomolecules, which regulate the host innate immune signaling pathways and the viral antagonism of host innate immune responses, could be used as potential antiviral drug targets. The red, blue, purple, peacock blue lines respectively indicate that IAV proteins, microRNAs, lncRNAs, vtRNAs target various steps of host innate immune signaling pathways.

**Figure 3.**
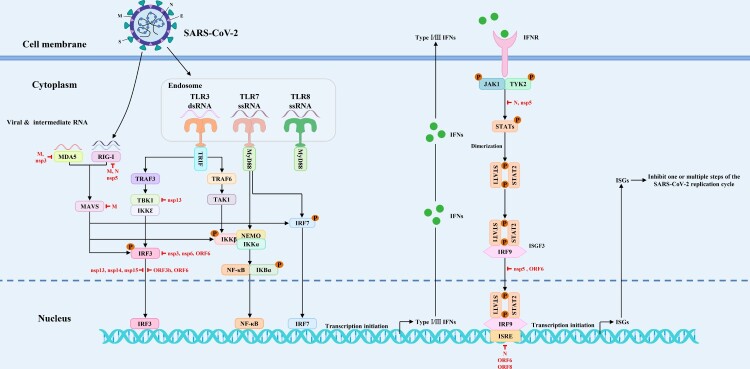
The mapping of antiviral drug targets focusing on SARS-CoV-2 antagonism of host innate immune signaling pathways. On the one hand, SARS-CoV-2 infection activates the host innate immune signaling pathways, thereby promoting the expression of type I/III interferons and downstream ISGs. On the other hand, SARS-CoV-2 can suppress the expression of interferons and downstream ISGs by targeting and inhibiting PRRs, signaling proteins, transcription factors, and interferon-activated JAK/STAT signal transduction. Theoretically, the viral antagonism of host innate immune signaling pathways could be used as potential antiviral drug targets. The red lines indicate that SARS-CoV-2 proteins target various steps of host innate immune signaling pathways.

In conclusion, in this review, we updated the IAV-host interaction network and SAR-CoV-2-host interaction network to better understand the biological pathogenesis of influenza and COVID-19. Besides, we proposed new strategies for designing antiviral drugs based on the virus-host interaction networks, which is expected to solve the problem of viral drug resistance. We firmly believe that the mappings of antiviral drug targets (Figures 1–3) focusing on the viral replication cycle or antiviral innate immune signaling pathways could provide richer potential drug targets and a theoretical basis for new strategies to design antiviral drugs based on virus-host interaction networks from the perspectives of viral immunology and systems biology. In particular, the virus-host interaction networks harbour three kinds of main antiviral drug targets: viral targets, host targets, and viral antagonism of host innate immunity, with viral targets being the traditional antiviral drug targets, host targets being selected as a secondary consideration, and viral antagonism of host innate immunity being the most overlooked in the antiviral drug development process.

## The IAV replication cycle

Theoretically, each of these steps in the viral replication cycle could be used as an antiviral drug target [11,12]. The complete IAV replication cycle comprises four stages: attachment, entry and uncoating, replication and transcription, assembly and release [22]. The hemagglutinin binds to sialic acid receptors on cells to mediate viral entry. The vRNPs are released into the cytoplasm after endocytosis by fusing the viral membrane with the cell endosomal membrane. Subsequently, the vRNPs enter the nucleus, where new vRNPs are synthesized with the viral mRNAs. Then these mRNAs are exported to the cytoplasm for translation of essential viral proteins. In the course of viral replication, newly synthesized polymerase proteins (PA, PB1, and PB2) and NP are imported into the nucleus, where they are assembled into vRNPs to support late transcription. Finally, newly synthesized structural components, including vRNPs, are transported to the cell membrane, where progeny viruses are assembled and formed by budding. A schematic diagram of the IAV replication cycle is shown in Figure 1.

## Host innate immune responses against IAV: fundamental components of the interaction networks

The host innate immune responses are the first line of defense against IAV infection, which includes three main phases: interferon production, expression of ISGs, and antiviral action of ISGs. In addition, some host restriction factors independent of interferon induction are also involved in regulating IAV replication. Thus, both host innate immune responses and host restriction factors, which regulate IAV replication, could be used as potential antiviral drug targets for the treatment of IAV infection. A schematic diagram of host innate immune responses against IAV is shown in Figure 2.

### Interferon induction

As a pattern recognition receptor in host cells, the RIG-I recognizes ssRNAs and dsRNAs produced during IAV replication in the cytoplasm. In addition, the MDA5 is responsible for the recognition of dsRNAs in the cytoplasm [23]. After IAV infection, RIG-I and MDA5 can recognize vRNAs and recruit MAVS to activate the NF-κB and IRF3/IRF7. These activated transcription factors enter the nucleus and bind promoter sites of the corresponding genes to induce the expression of cytokines such as type I and type III interferons. It has been reported that TRIM25, Riplet, and PACT regulate RIG-I-mediated type I interferon induction [24–26]. Besides, TLR3 is responsible for recognizing dsRNAs, while TLR7/8 is responsible for recognizing ssRNAs in endosomes [2]. Upon recognition, TLR3 interacts with the adaptor protein TRIF for subsequent activation of NF-κB and IRF3, while TLR7 acts on the adaptor protein MyD88 for subsequent activation of NF-κB and IRF7 [2]. During this process, MAVS, TRAF3, TRAF6, TAK1, IκBα, TBK1/IKKϵ, and IKKα/IKKβ/NEMO complexes act as essential signaling proteins to play a signal transduction role.

### Expression of ISGs

Type I and III interferons, induced by activated transcription factors NF-κB and IRF3/IRF7, bind to their corresponding receptors on target cells in an autocrine or paracrine manner, thus inducing the phosphorylation of JAK1 and TYK2 kinases, which further induces downstream STAT1 and STAT2 phosphorylation. Upon phosphorylation, STAT1 and STAT2 join with IRF9 to form the transcription factor complex ISGF3. Subsequently, the ISGF3 trimers migrate to the nucleus and bind ISRE to initiate the expression of ISGs that can ultimately inhibit the IAV replication [2].

### Antiviral action of ISGs and other host restriction factors

ISGs are one of the host restriction factors that can directly or indirectly inhibit viral replication in host cells and can be expressed to inhibit viral replication under interferon stimulation, while other host restriction factors can exert antiviral effects independent of interferon responses [27]. It has been found that the host restriction factors can block one or multiple steps of the IAV replication cycle, which is composed of four stages: attachment, entry and uncoating, replication and transcription, assembly and release. Therefore, these host restriction factors could provide many potential antiviral drug targets for the treatment of IAV infection. Roles of host restriction factors that inhibit IAV replication are shown in Figure 1 and Table 1.

**Table 1.** Roles of host restriction factors that inhibit IAV replication.

Categorization		The host restriction factors
The host restriction factors that directly inhibit IAV replication by regulating the IAV replication cycle	Attachment	B4GALNT2, SPOCK2
	Entry	CK2β, FGFR1, NCOA7, Nrf2, SPOCK2
	Endosomal fusion and release	IFITM3
	vRNPs import	BPIFA1, CK2β, MOV10, MxA, PLSCR1
	Replication	ISG20, NCL, STAT1, TRIM56, YBX3
	Proteins import	eEF1D, MxA
	Assembly of RNA polymerase	DDX21, SERTAD3
	Polymerase activity	HDAC6, hnRNP-F, ISG20 NCL, PACT, PKP2, RTF2
	Assembly of vRNPs	CypE, RBL2
	vRNPs export	SOD1
	Transcription	Mx1, RTF2, TRIM25
	mRNAs export	hnRNP A2/B1, Nup98, Rae1
	Protein synthesis	MxA, ZAPS, ZFP36L1
	Post-translational modification of proteins	HDM2, ISG15
	Degradation	CypA, Grail, p27Kip1, TRIM14, TRIM22 TRIM32, TRIM35, TRIM41, USP11, ZAPL
	Protein transport	ARHGAP21
	Assembly of virion	CyclinD3
	Budding	AnxA6, Viperin
	VLP budding	Tetherin
	Release	AnxA6
The host restriction factors that indirectly inhibit IAV replication by regulating the innate immune signaling pathways		AGO4, Dot1L, eIF4B, FBXW7, FGF2, FKBP5, GBP5, HDAC2, HDAC4, HDAC11, HERP, HIST1H1C, MCCC1, MVP, NF90, NLRC5, NMB, p27Kip1, PACT, RSK2, RTF2, S1PR1, SLFN14, SNW1, SPL, TIP60, TRIM14, TRIM35, ZMPSTE24

## Supportive host factors that promote IAV replication: fundamental components of the interaction networks

The frequent evolution of viruses not only changes the severity of the disease but also contributes to the development of drug resistance. In this regard, the modulation of host factors involved in regulating viral replication is a good strategy against viral diseases [11,12]. Host factors related to IAV replication include host restriction and supportive host factors. Supportive host factors can directly or indirectly promote viral replication. Studies have shown that IAV can hijack host translation systems, thereby promoting the expression of supportive host factors to meet their needs [5,28]. Roles of supportive host factors that promote IAV replication are shown in Figure 1 and Table 2.

**Table 2.** Roles of supportive host factors that promote IAV replication.

Categorization		The supportive host factors
The supportive host factors that directly promote IAV replication by regulating the IAV replication cycle	Attachment	Akt
	Entry	Akt, EGFR, FAK, FFAR2, GBA, LY6E MCOLN2, PLC-γ1, PLD2, RNASEK, SLC35A1
	Endosomal fusion and release	ACP2, CtsW, EPS8, GRK2, Itch, PEPD
	vRNPs import	ACP2, AP2M1, Hsp40, SH3GLB1
	Replication	IREF-2, JNK, PRPF8, α-actinin-4
	Polymerase activity	ANP32A, ANP32B, ATP7A, CDC25B, CHD1, CLE, CTR1, DDX17, DR1, eIF4A3, GPS1, HMGB1, PKC, PPP6C
	Assembly of vRNPs	FMRP, LYAR
	vRNPs export	CD151, CHD3, eIF4A3, Hsc70, KIF18A MLC, NOX2, Nucleolin, PRC2, RanBP3, SGK1, SK, TrkA, XPO1
	Transcription	CDK9, ERI1, RRP1B
	mRNAs export	DDX19, eIF4A3, NUP93, ZC3H11A
	Protein synthesis	eEF1G, JNK, P58IPK
	Post-translational modification of proteins	AIMP2, CNOT4, CRL4
	Protein transport	Cdc42, NUMA1, UBR4
	Assembly of virion	Cofilin-1
	Budding	Cofilin-1, F1β
	Release	NOX2, RACK1
The supportive host factors that indirectly promote IAV replication by regulating the innate immune signaling pathways		A20, AGO2, CUEDC2, DPF2, DR1, EZH2, FADD, FAT10, GLDC, GPS1, IFI44L, KHSRP, MK2, MK3, MKP5, NDRG1, NEDD4, PARP1, PGRN, PLD2, Rubicon, SK, SOCS1, SOCS3, TAP1, TRIM21, USP7, ZBTB25

## ncRNAs involved in regulating IAV replication: fundamental components of the interaction networks

It has been recognized that ncRNAs are novel regulators participating in various cell biological processes, such as cell growth, differentiation, survival, and apoptosis. In addition to the host factors mentioned above, it has been demonstrated that ncRNAs regulate IAV replication, such as microRNAs, lncRNAs, circRNAs, and vtRNAs. Therefore, the systematic understandings of the ncRNAs involved in regulating IAV replication and their regulatory mechanisms are essential for exploring antiviral drug targets.

### MicroRNAs

Studies have shown that microRNAs can affect IAV replication by directly regulating the expression of viral proteins. Besides, they can indirectly affect IAV replication through various mechanisms by targeting and regulating PRRs, signaling proteins, transcription factors, JAK-STAT signaling pathway, host restriction factors, and supportive host factors. First and foremost, miR-3145, miR-323, miR-324-5p, miR-485, miR-491, and miR-654 inhibit PB1 expression [29–32]; miR-1249, miR-188-3p, and miR-584-5p inhibit PB2 expression [33,34]; miR-1307-3p inhibits NS1 expression [35]; and miR-let-7c inhibits M1 expression [36]. In addition, miR-136, miR-340-5p, and miR-485 target and regulate the expression of PRRs such as RIG-I [32,37,38]. Furthermore, miR-125a, miR-125b, miR-144, miR-146a, miR-194, miR-302c, miR-4776, and miR-93 target and regulate the expression of signaling proteins MAVS, MAVS, TRAF6, TRAF6, TBK1, NIK, NFKBIB, and JAK1, respectively [6, 39–42]. What's more, miR-30 targets and regulates the expression of SOCS1 and SOCS3. Additionally, miR-132-3p, miR-144, miR-194, and miR-302a regulate the expression of transcription factors IRF1, IRF7, IRF3, and IRF5, respectively [40,41]. For supportive host factors, miR-1-3p, miR-17-3p, miR-193b, miR-203, miR-221, miR-26a, miR-30, miR-324-5p, miR-33a, and miR-548an respectively inhibit the expression of ATP6V1A, GALNT3, β-catenin, DR1, GALNT3, USP3, NEDD4, CUEDC2, ARCN1, and NS1ABP to inhibit IAV replication ultimately, but miR-34c promotes PLK4 expression to promote IAV replication. For host restriction factors, miR-1290, miR-194, miR-21-3p, miR-340-5p, miR-4276, miR-664a-3p, and miR-9 respectively inhibit the expression of VIM, FGF2, HDAC8, OAS2, COX6C, LIF, and MCPIP1 to ultimately promote IAV replication [38,41]. Notably, miR-144, miR-194, miR-30, miR-324-5p, and miR-340-5p can regulate the IAV replication through multiple mechanisms, which deserve more attention in the selection process of antiviral drug targets. Besides, Harshad Ingle et al. uncovered that infection of various human and mouse cells with RNA viruses, including the H5N1 influenza virus, resulted in the increased production of the microRNA miR-485, which targeted RIG-I mRNA for degradation. As a result, antiviral signaling was inhibited and viral replication was enhanced. However, when cells were exposed to increased amounts of virus, miR-485 was expressed, but viral replication was inhibited. These findings highlight the dual role of miR-485 in preventing activation of antiviral signaling and restricting influenza virus infection [32]. Therefore, as a host target, the dual role of miR-485 in regulating IAV replication should be extensively considered in the selection process of anti-IAV drug targets, otherwise, there is a risk of drug side effects. Roles of microRNAs involved in regulating IAV replication are shown in Figures 1 and 2, and Table 3.

**Table 3.** Roles of microRNAs involved in regulating IAV replication.

microRNAs	Targets	Effect on IAV replication	Expression level after IAV infection	IAV subtypes	Cell lines
miR-1249	PB2, ↓	Inhibitive	↓	H5N1	A549
miR-125a, miR-125b	MAVS, ↓	Promotive	↑	H1N1, H3N2	pBECs
miR-1290	VIM, ↓	Promotive	↑	H1N1	A549
miR-1307-3p	NS1, ↓	Inhibitive	↓	H1N1	A549
miR-132-3p	IRF1, ↓	Promotive	↑	H1N1	A549
miR-136	RIG-I, ↑	Inhibitive	N/A	H5N1	A549
miR-1-3p	ATP6V1A, ↓	Inhibitive	↓	H1N1, H3N2	A549
miR-144	TRAF6, ↓ IRF7, ↓	Promotive	N/A	H1N1	murine lung epithelial cells
miR-146a	TRAF6, ↓	Promotive	↑	H1N1	A549
miR-155	N/A	Inhibitive	N/A	H1N1	N/A
miR-17-3p	GALNT3, ↓	Inhibitive	↓	H1N1, H3N2	A549
miR-188-3p	PB2, ↓	Inhibitive	N/A	H1N1, H5N6, H7N9	A549
miR-193b	β-catenin, ↓	Inhibitive	↓	H1N1	A549, HEK293
miR-194	Phosphorylation of TBK1 and IRF3, ↓ FGF2, ↓	Promotive	↓	H1N1	A549, HEK293T
miR-203	DR1, ↓	Inhibitive	↑	H5N1	A549
miR-21-3p	HDAC8, ↓	Promotive	↓	H1N1, H5N1	A549
miR-21-3p	FGF2, ↓	Promotive	↓	H5N1	A549
miR-221	GALNT3, ↓	Inhibitive	↓	H1N1, H3N2	A549
miR-26a	USP3, ↓	Inhibitive	↓	H1N1	A549, HEK293T
miR-30	SOCS1,SOCS3, NEDD4, ↓	Inhibitive	↓	H5N1	A549
miR-302a	IRF5, ↓	Inhibitive	↓	H1N1	A549, PBMC
miR-302c	NIK, ↓	Promotive	↓	H3N2	A549
miR-3145	PB1, ↓	Inhibitive	N/A	H1N1, H3N2, H5N1	A549
miR-323	PB1, ↓	Inhibitive	↑	H1N1	MDCK, HEK293T
miR-324-5p	CUEDC2, ↓ PB1, ↓	Inhibitive	↓	H1N1, H5N1	A549, SAEC
miR-33a	ARCN1, ↓	Inhibitive	↓	H1N1, H3N2, H9N2	A549, HEK293T, Hela
miR-340-5p	RIG-I, ↓ OAS2, ↓	Promotive	↓	H5N1	A549
miR-34c	PLK4, ↑	Promotive	↑	H1N1	A549
miR-4276	COX6C, ↓	Promotive	↓	H1N1, H3N2	A549
miR-4776	NFKBIB, ↓	Promotive	↑	H1N1	HBEpC
miR-485	RIG-I, ↓	Promotive	↑	H5N1	HEK293T
	PB1, ↓	Inhibitive	↑	H5N1	HEK293T
miR-491	PB1, ↓	Inhibitive	↓	H1N1	MDCK, HEK293T
miR-548an	NS1ABP, ↓	Inhibitive	↓	H1N1	A549
miR-584-5p	PB2, ↓	Inhibitive	↓	H5N1	A549
miR-654	PB1, ↓	Inhibitive	↑	H1N1	MDCK, HEK293T
miR-664a-3p	LIF, ↓	Promotive	↑	H3N2, H7N9	A549
miR-9	MCPIP1, ↓	Promotive	↑	H1N1, H3N2	A549
miR-93	JAK1, ↓	Promotive	↓	H1N1	AT2, MLE-12
miR-let-7c	M1, ↓	Inhibitive	↑	H1N1	A549

### LncRNAs

It has been shown that lncRNAs can regulate the antiviral innate immune responses at the transcriptional, translational, and chromatin levels. Moreover, lncRNAs can also affect IAV replication by protecting viral proteins from degradation, regulating host cell metabolism, and increasing the RNA polymerase activity. For example, lncRNA-155, lncRNA-ISG20, lncRNA-MxA, and lncRNA-TSPOAP1-AS1 can target and regulate the expression of interferons and downstream ISGs at the transcription and translation levels [43–46], while lncRNA-IVRPIE and lncRNA-NRAV target and regulate it at chromatin levels [7,47]. Besides, lncRNA-IPAN can protect the viral proteins from degradation [48], while lncRNA-PAAN can increase the RNA polymerase activity [49]. Interestingly, lncRNA-ACOD1 can affect IAV replication by regulating host cell metabolism [50]. These lncRNAs involved in regulating IAV replication enrich the variety of host antiviral drug targets in interaction networks. Roles of lncRNAs involved in regulating IAV replication are shown in Figures 1 and 2, and Table 4.

**Table 4.** Roles of lncRNAs involved in regulating IAV replication.

LncRNAs	Effect on IAV replication	Expression level after IAV infection	Mechanisms
lncRNA-155	Inhibitive	↑	Inhibits the expression of PTP1B, a negative regulator of the interferon signaling pathway, to promote the production of IFN-β and ISGs, such as IFITM3
lncRNA-ACOD1	Promotive	↑	binds directly to Glutamate oxalate transaminase-2 (GOT2), thereby promoting the catalytic activity of GOT2 and the synthesis of its metabolites to facilitate IAV replication
lncRNA-IPAN	Promotive	↑	Binds to PB1 protein to form the IPAN/PB1 complex, thereby protecting PB1 from degradation
lncRNA-ISG20	Inhibitive	↑	As a ceRNA, it counters the targeting inhibitory effect of miR-326 on ISG20 by binding to miR-326, promoting ISG20 translation indirectly
lncRNA-IVRPIE	Inhibitive	↑	Affects histone modifications at the transcriptional start site of IFNβ and ISGs, and promotes the expression of ISGs, such as ISG15
lncRNA-MxA	Promotive	↑	Blocks the transcription of IFN-β through the formation of RNA-DNA triplex structures at the promoter site, thereby inhibiting the expression of ISGs, such as IFITM3
lncRNA-NRAV	Promotive	↓	affects histone modifications at the transcription start site and suppresses the expression of ISGs, such as IFITM3
lncRNA-PAAN	Promotive	↑	Interacts with PA protein to promote the assembly of RdRp complexes, thus increasing the RNA polymerase activity
lncRNA-TSPOAP1-AS1	Promotive	↑	Inhibits transcription of IFN-β and activation of the ISRE promoter, thereby suppressing the expression of ISGs, such as IFITM3

### CircRNAs and vtRNAs

Aside from microRNAs and lncRNAs, it has been reported that circRNAs and vtRNAs regulate IAV replication. Tianqi Yu et al. found that H1N1 IAV infection-induced circRNA-GATAD2A expression promotes IAV replication by inhibiting autophagy [8]. Interestingly, Zhiyuan Qu et al. have identified a circRNA, which could antagonize influenza virus by absorbing a microRNA that degrades CREBBP and accelerating IFN-β production [51]. Additionally, Fang Li et al. uncovered that vtRNAs could regulate the IAV replication at cellular and animal levels. Further studies revealed that vtRNAs induced by a viral infection could promote the IAV replication by inhibiting PKR activation and the subsequent interferon expression [9].

## Other host biomolecules involved in regulating IAV replication: fundamental components of the interaction networks

Aside from proteins and nucleic acids, studies have shown that sugars, lipids, hormones, and inorganic salts also affect IAV replication by regulating the viral replication cycle or innate immune signaling pathways, which could also be used as antiviral drug targets. Specifically, glucose is the sugar that promotes IAV replication [52]. For lipids, SM is found to facilitate IAV replication, while ceramide, cholesterol, and PD1 act as inhibitors [53]. For hormones, glucocorticosteroids positively affect IAV replication, but oestrogenic hormones are inhibitory [54]. For inorganic salts, Zn is observed as a positive regulator for IAV replication [55]. Interestingly, Zhuo Luo et al. showed that the CORT level was increased significantly under stress conditions, thereby ultimately mediating the degradation of MAVS to promote IAV replication [56]. CORT is the major type of glucocorticoids, so this work connected hormones to innate immune signaling pathways and enriched our understanding of biomolecular regulatory networks.

## IAV antagonism of host innate immunity: fundamental components of the interaction networks

Studies indicated that the virus-host interaction networks harbour three kinds of main antiviral drug targets: viral targets, host targets, and viral antagonism of host innate immunity, with viral targets being the traditional antiviral drug targets, host targets being selected as a secondary consideration, and viral antagonism of host innate immunity being the most overlooked in the antiviral drug development process [17,57]. IAV had evolved multiple strategies to antagonize host innate immunity, such as blocking various steps of host innate immune signaling pathways or regulating microRNAs expression and lipid synthesis [5,33,53]. These mechanisms affect gene expression at the transcriptional, translational, post-translational modification, and epigenetic levels.

### Block host innate immune signaling pathways

It has been demonstrated that IAV can block the host innate immune signaling pathways by several strategies. First and foremost, IAV could suppress the expression of interferons and downstream ISGs by targeting and inhibiting PRRs, signaling proteins, transcription factors, interferon promoter activity, and interferon-activated JAK/STAT signal transduction. Shi Liu et al. showed that IAV NS1 proteins could regulate the expression of some key regulators of JAK-STAT signaling by inhibiting the DNA methylation of their promoters so as to antagonize host innate immunity. This research revealed a new strategy of IAV to antagonize host innate immunity by epigenetic modifications [10]. In addition, IAV can also inhibit the interferon-mediated JAK-STAT signaling pathway by inducing SOCS1 and SOCS3 expression. For example, SOCS1 participates in the degradation of JAK1 [28], while SOCS3 inhibits the phosphorylation of STAT1 and STAT2 [5].

Interestingly, strategies of IAV to antagonize host innate immunity are characteristic in three aspects. To begin with, multiple viral proteins can work together to antagonize one host factor. Additionally, one viral protein can antagonize multiple host factors or signal transduction processes. What's more, IAV can reduce the content of one host factor at multiple levels of gene expression regulation, including mRNA transcription and protein degradation. A schematic diagram of strategies of IAV to antagonize host innate immune signaling pathways are shown in Figure 2 and Table 5. Notably, NS1 is encoded by all strains of IAV, serving as a key protein in the “game” between IAV and host in innate immunity. Particularly, NS1 could antagonize multiple signal transduction processes at the transcriptional, translational, post-translational modification, and epigenetic levels, making it a valuable target for developing antiviral drugs [10]. More specifically, these drugs may bind to NS1 or interfere with the interactions between NS1 and its cellular targets to exert their efficacy. Scholars have achieved consensus about the feasibility of this idea for antiviral drug development [57].

**Table 5.** Strategies of IAV to antagonize host innate immune signaling pathways.

Classification of targets or other signal transduction processes	Targets	Viral proteins	Mechanisms
PRRs	RIG-I	NS1	Inhibits signal transduction mediated by targets and thus down-regulate the expression of interferons and downstream ISGs
Signaling proteins	MAVS	PB1-F2	
		PB2	
	TRAF3	NS1	
		PB2	
	IKKα	NS1	
	IKKβ	NS1	
		PB1-F2	
Transcription factors	NF-κB	NS1	
	IRF3	NS1	
		PA	
The nuclear import of transcription factors	IRF3	PA	
	NF-κB	PA-X	
The transcription of interferon	promoter regions	NS1	Blocks IFN-β transcription and thereby inhibits the expression of interferons and downstream ISGs

### Regulate microRNAs expression and lipid synthesis

It is noteworthy that previous studies found many microRNAs can directly or indirectly suppress IAV replication, including miR-1249, miR-1307-3p, miR-1-3p, miR-17-3p, miR-193b, miR-221, miR-26a, miR-30, miR-302a, miR-324-5p, miR-33a, miR-491, miR-548an and miR-584-5p (Table 3). Interestingly, the expression of these microRNAs was down-regulated in the early stage of IAV infection. Similarly, Morita et al. reported that PD1 could also inhibit IAV replication by suppressing the nuclear export of vRNAs. In a further study, they found that the synthesis of PD1 was significantly inhibited when infected with IAV. These studies help to further expand the targets of IAV antagonism from host factors to ncRNAs and lipids [53].

### IAV-host interaction networks: current research trends and future directions

To accelerate the study's progress on virus-host interaction networks, we taked IAV as representatives of viruses and discuss the current research trends and future directions on virus-host interaction networks. Currently, studies on IAV-host interaction network mainly focus on the IAV replication cycle and host innate immune responses. Therefore, we updated the mappings of antiviral drug targets focusing on IAV replication cycle or host innate immune responses against IAV, which could provide richer potential drug targets and a theoretical basis for new strategies to design antiviral drugs based on virus-host interaction networks from the perspectives of viral immunology and systems biology. The mapping of antiviral drug targets focusing on IAV replication cycle is shown in Figure 1. The mapping of antiviral drug targets focusing on host innate immune responses against IAV is shown in Figure 2.

### Identification of host biomolecules involved in regulating IAV replication

In recent years, high-throughput sequencing techniques, such as transcriptome sequencing, microRNA sequencing, and proteomics have been widely used in various fields of life sciences [7,51]. Given this, future studies can continue to integrate interference, overexpression, and high-throughput sequencing techniques together to identify more biomolecules involved in regulating IAV replication and broaden the scope of potential antiviral drug targets in the virus-host interaction networks. For example, several hundred circRNAs, which show up-regulation or down-regulation after IAV infection, may be obtained from sequencing results, and the top 50–100 circRNAs with high differences in expression should be given special attention as they could play a more important biological role during IAV infection. Following that, RNA interference and overexpression techniques could be used to identify the specific circRNAs involved in regulating IAV replication [51].

### Regulatory mechanisms of IAV replication and biomolecular regulatory networks

To explore antiviral drug targets, the systematic understandings of the regulatory mechanisms of IAV replication and biomolecular regulatory networks are essential. After IAV infection, host biomolecules such as host factors, ncRNAs, sugars, lipids, hormones, and inorganic salts co-regulate IAV replication and form biomolecular regulatory networks. In the first place, we try to discuss the regulatory mechanisms of IAV replication. The molecular mechanisms regulating IAV replication by ncRNAs have gradually become a research hot spot in recent years. Many studies have been done regarding the issue of whether these ncRNAs can directly affect IAV replication by regulating the IAV replication cycle or indirectly affect IAV replication by regulating the innate immune signaling pathways [7,43,48,49]. In particular, special attention has been paid to the expression of which host factors these ncRNAs can regulate to affect IAV replication ultimately [7,43].

Next, we intend to discuss the regulatory relationships among host biomolecules. Current studies mainly focus on the upstream-downstream relationships among the same class of host biomolecules and the regulatory relationships among different classes of host biomolecules [6,7,9,50]. Currently, we don't know which PRRs, adaptor proteins, signaling proteins, and transcription factors are required to express the majority of the host factors, microRNAs, and lncRNAs mentioned above. Consequently, we propose that TLR3, TLR7, or other knockout mice can be constructed by CRISPR/Cas9 technology to investigate the upstream-downstream relationships among the same class of host biomolecules. In addition, studies have reported that some host restriction factors, supportive host factors, microRNAs, and lncRNAs belong to ISGs [44]. Therefore, interferon receptor knock-out mice can be constructed to systematically explore which of the host factors or ncRNAs mentioned above belong to ISGs. It is worth noting that some activated signaling pathways, such as JNK-AP-1, can promote IAV replication. Similarly, we can systematically explore which of the above-mentioned supportive host factors is expressed in dependence on the activation of JNK-AP-1. Interestingly, Wenjia Chai et al. reported that lncRNA-ISG20 could indirectly regulate the IAV replication by regulating other ncRNAs, such as miR-326 [44]. Excitingly, Pin Wang et al. revealed a new mechanism that IAV can exploit lncRNA-ACOD1 to regulate host cell metabolism to favour IAV replication. The research results provide a new idea about the regulatory mechanisms among different classes of host biomolecules [50]. In short, we can further refine the regulatory relationships among these biomolecules by multi-omics joint analysis. Besides, it deserves thoughtful consideration whether the key biomolecules in biomolecular regulatory networks can be screened. If so, it is bound to lay an extremely important theoretical foundation for designing antiviral drugs based on multiple host targets. Recently, researchers began to realize that virus-infected cells can regulate the innate immune responses in uninfected neighbouring cells by exosome-mediated signal transmission [58]. These studies lay the foundation for a further understanding of biomolecular regulatory networks based on intercellular association.

Finally, we will discuss the research ideas about the regulatory mechanisms among host biomolecules. Based on the current research ideas about IAV-host interactions, we only introduce protein–protein interactions, classified into three types: host-host protein interactions, virus-host protein interactions, and virus-virus protein interactions. The research ideas about protein–protein interactions mainly focus on determining upstream-downstream relationships, interacting domain and even binding site, and subcellular localization [59]. It is worth mentioning that the network of all interactions among proteins in the living body is called “protein–protein interaction networks”. Some researchers have proposed the construction methods of dynamic protein–protein interaction networks, which have contributed to the study of dynamic biological processes such as the occurrence and development of various diseases [60]. In conclusion, we strongly believe the in-depth study of regulatory mechanisms of viral replication and biomolecular regulatory networks could accelerate the development of antiviral drugs based on virus-host interaction networks.

### Mechanisms of the innate immune responses against IAV at the animal level or single-cell level

Rigorous identifications of gene functions and the antiviral drug targets require validation at the animal level. However, most research results are obtained at the cellular level, especially those of microRNAs and lncRNAs involved in regulating IAV replication. In recent years, point mutant mice have been constructed to investigate the biological functions of protein phosphorylation modification. This approach lets us screen the key amino acid sites of IRF3, IFR7, and other proteins and explore the molecular mechanisms that host factors, especially transcription factors, regulate IAV replication after phosphorylation by multi-omics joint analysis. Notably, traditional studies on host innate immune responses are generally limited to cellular or animal levels, obscuring the differences and connections between different cell lines. To overcome such obstacles, Yael Steuerman et al. used single-cell sequencing and found heterogeneous properties of viral loads among nine cell types in the lung [61]. Interestingly, Irene Ramos et al. used single-cell sequencing and found that type III interferon played a key role in paracrine signaling after IAV infection [62]. These studies profoundly elucidated the interaction relationships between IAV and host at the single-cell level, thereby providing different targets for designing new antiviral drugs based on virus-host interaction networks.

## Virus-host interaction networks as new antiviral drug targets for IAV

The potential antiviral drug targets are important for developing effective antiviral drugs. Most approved antiviral drugs target a viral enzyme that plays an essential role in viral replication. In addition, host cell pathways and virus-host interactions are being used as antiviral targets [11–13]. To date, only two classes of antiviral drugs are globally approved and available for the treatment of influenza infections: M2 ion channel blockers and NA inhibitors. The first class includes adamantane derivatives, amantadine, and rimantadine, which inhibit proton conductivity of the M2 ion channel of IAV, hence preventing the viral uncoating step of the viral replication cycle. However, because of viral recombination or drug resistance mutations under the drug selection pressure, current anti-IAV drugs have difficulty coping with the constantly mutating viruses, and the vaccines often fail in disease prevention. Therefore, drug resistance is a considerable challenge in antiviral therapy, and the design of new anti-IAV drugs has been urgent in clinics. Viruses rely on host cellular functions to replicate, and therefore a thorough understanding of the roles of virus-host interaction networks during IAV replication is essential to develop new anti-IAV drugs [16]. Recently, IAV-host interactions have been widely investigated, which deepened the understanding of the host biomolecules involved in regulating IAV replication and the molecular mechanisms of IAV to antagonize host innate immunity. Host biomolecules are not easy to mutate as virus proteins are, so all biomolecules involved in various steps of the IAV replication cycle or even the innate immune signaling pathways could be considered as the drug targets theoretically. It needs to be emphasized that antiviral drugs targeting host biomolecules do not act directly on viruses but regulate the biochemical process in the host cells. Besides, due to the relatively few research results of IAV to antagonize host innate immunity, the interactions, including protein–protein and protein-RNA, between viral proteins and host biomolecules were often ignored when designing traditional drugs in the past 10 years. Therefore, innovative insights will be provided for the design of new anti-IAV drugs by an in-depth study on interactions between viral proteins and host biomolecules with the help of structural biology. In conclusion, the race between host antiviral innate immunity and viral antagonism of host innate immunity forms virus-host interaction networks, which harbour abundant potential antiviral drug targets. Given this, we updated the mappings of antiviral drug targets (Figures 1–3) and proposed new strategies to design anti-IAV drugs based on the virus-host interaction networks from the perspectives of viral immunology and systems biology, which is expected to solve the problem of viral drug resistance. The IAV-host interaction networks harbour three kinds of main antiviral drug targets: viral targets, host targets, and viral antagonism of host innate immunity. To begin with, viral targets are the traditional antiviral drug targets. Theoretically, each of these steps in the IAV replication cycle could be used as a potential antiviral drug target. Additionally, these host biomolecules, which regulate the IAV replication cycle and host innate immune signaling pathways, are selected as a secondary consideration. Last but not least, IAV antagonism of host innate immunity in interaction networks could also be used as potential antiviral drug targets, which are the most overlooked in the antiviral drug development process.

Unlike the development strategies of new antiviral drug targets for IAV proposed by some scholars, we summarized a more comprehensive understanding of the IAV-host interaction networks from the perspectives of viral immunology and systems biology, providing richer potential drug targets and a theoretical basis for developing new anti-IAV drugs based on the IAV-host interaction networks [16–18]. Initially, when exploring the potential drug targets in the hosts, previous studies mainly focused on proteins involved in regulating IAV replication, such as host restriction factors, supportive host factors, receptors, and enzymes. However, this article also systematically discussed microRNAs, lncRNAs, circRNAs, vtRNAs, sugars, lipids, hormones, and inorganic salts. Studies have shown that these host biomolecules can directly affect IAV replication by regulating the IAV replication cycle or indirectly affect IAV replication by regulating the innate immune signaling pathways. Furthermore, previous studies mainly focused on the viral replication cycle, while the innate immune responses against IAV were summarized from the perspectives of viral immunology and systems biology in this article, establishing a theoretical foundation to design new anti-IAV drugs based on innate immune signaling pathways. Besides, previous explorations on the potential host drug targets depended on bioinformatics analysis, which relatively lacked crucial experimental verification. However, the potential drug targets summarized in this paper were proposed from experimental reports, providing a strong guarantee for developing new anti-IAV drugs [16,17]. Furthermore, multiple molecular mechanisms of IAV to antagonize host innate immunity were summarized in this paper, providing innovative insights for designing new anti-IAV drugs targeting the interactions between viral proteins and host biomolecules.

In recent years, traditional ideas of developing new anti-IAV drugs based on single targets have gradually shown their limitations. When treating complex diseases, traditional antiviral drugs are often incapable of producing the desired effects. With the development of proteomics and system biology, researchers started to integrate biological networks and drug-target networks to analyze the effect of drugs on the crucial nodes or other biomolecules in the overall biological networks. Thus, the searching targets have switched from single to network targets, resulting in a new discipline named network pharmacology. Given this, we can make full use of network pharmacology, structural biology, system biology, and computer-aided design to develop new anti-IAV drugs based on multiple targets [63]. In conclusion, we have every reason to believe that the combination of new and traditional anti-IAV drugs, which are called therapeutic cocktails in the future, would possess broad-spectrum anti-IAV activities to counter the potential influenza pandemic as a result of viral mutation.

## Virus-host interaction networks as new antiviral drug targets for SARS-CoV-2

### SARS-CoV-2-host interaction networks as new antiviral drug targets

Since the outbreak of the COVID-19 pandemic, it has spread rapidly around the world. So far, about 400 million people have been infected worldwide, and about six million have died, seriously threatening global public health and economic development. As of yet, no specific antiviral drug has been discovered for SARS-CoV-2. Therefore, there is an urgent need to explore antiviral drug targets for SARS-CoV-2 and develop effective clinical drugs. This requires a deeper understanding of the SARS-CoV-2 replication cycle and the SARS-CoV-2-host interaction networks to provide a richer theoretical basis for developing anti-SARS-CoV-2 drugs. As a member of the Coronaviridae family, SARS-CoV-2 is an enveloped, single-stranded positive-sense RNA virus, which can encode 16 non-structural proteins (nsp1-16), 4 structural proteins (E, M, N, S), and 9 accessory proteins (ORF3a, 3b, 6, 7a, 7b, 8, 9b, 9c and 10) [64]. Among them, the SARS-CoV-2 polymerase complex is composed of a catalytic subunit nsp12 and its cofactors nsp7-nsp8, which contribute to its important regulatory role in genome replication and other processes [65]. Currently, the studies on SARS-CoV-2-host interaction network are still in its initial stage. Therefore, the research results of SARS-CoV-2-host interaction network are summarized relatively systematically, which we hope will provide important inspirations for subsequent researchers and accelerate the study's progress in this field.

Host biomolecules, which mediate viral invasion or regulate viral replication, could serve as potential antiviral drug targets. Viral entry is the first step to infect host cells. Markus Hoffmann et al. showed that SARS-CoV-2 cell entry depends on ACE2 and TMPRSS2, which are the potential targets for antiviral intervention [66]. What's more, the viral antagonism of host innate immune signaling pathways could be used as potential antiviral drug targets theoretically. Since both SARS-CoV-2 and IAV have enveloped RNA viruses, their molecular mechanisms are similar. The mapping of antiviral drug targets focusing on SARS-CoV-2 antagonism of host innate immune signaling pathways is shown in Figure 3. It is worth mentioning that the research ideas of SARS-CoV-S are similar to that of IAV. By proteomics, Denisa Bojkova et al. found that some metabolic pathways regulate SARS-CoV-2 replication. Furthermore, They further confirmed that SARS-CoV-2 replication depends on nucleotides in host cells by administering compounds that can interfere with nucleotide metabolism, such as ribavirin [67]. Interestingly, by mass spectrometry-based proteomics, Mehdi Bouhaddou et al. systematically analyzed the phosphorylation modifications resulting from SARS-CoV-2 infection. They found that the inhibitors of CK2, p38 MAPK, PIKFYVE, and CDKs kinase family have great power to inhibit SARS-CoV-2 replication [68]. Additionally, Kevin Klann et al. found that SARS-CoV-2 infection can activate growth factor receptor (GFR) and downstream signaling pathways in the hosts by phosphorylation proteomics, and drugs targeting them can inhibit the SARS-CoV-2 replication [69]. In addition, some scholars attempted to understand the mechanisms of SARS-CoV-2-host interaction from an epigenetic perspective and proposed new strategies based on epigenetics for targeted drug therapy [70]. Excitingly, David E Gordon et al. cloned, labelled, and expressed 26 of 29 viral proteins in human cells and subsequently identified 332 highly plausible interactions between viral proteins and human proteins by affinity purification-mass spectrometry (AP-MS). Meanwhile, they found that SARS-CoV-2 can interact with multiple innate immune signaling pathways [64]. These research results of SARS-CoV-2-host interaction network could provide important references for the subsequent development of anti-SARS-CoV-2 drugs.

### Research ideas for SARS-CoV-2-host interaction network: important inspirations initiated from the research results of IAV-host interaction network

At present, the research ideas on the SARS-CoV-2-host interaction network are limited to the reference of previous research results on other coronaviruses, including SARS-CoV and MERS-CoV. However, these results have not yet developed into a relatively systematic theoretical foundation, thus resulting in the slow progress of SARS-CoV-2 study. As a result, we believe that our focus should be directed toward viruses that have been thoroughly studied. After multiple screenings, IAV stands out among other viruses. In comparing the characteristics of the two viruses, we found that there are many unexpected similarities between SARS-CoV-2 and IAV. First and foremost, both are enveloped single-stranded RNA viruses. In addition, both can cause respiratory tract infections. Besides, lung and A549 were the main target organs and cell models, respectively. Furthermore, after infection with IAV and SARS-CoV-2, RIG-I, MDA5, TLR3, TLR7, and other PRRs in the host can effectively recognize vRNAs of both viruses and activate transcription factors, including NF-κB, IRF3 and IRF7, and JAK-STAT signaling pathway to initiate the innate immune responses [71]. What's more, both can target and inhibit the PRRs, signaling proteins, transcription factors, and signal transduction mediated by interferon receptors to inhibit the expression of interferons and downstream ISGs, ultimately antagonizing host innate immunity. Additionally, Both can carry risks of cross-species transmission and cause acute lung injury and multiple organ failure, which seriously endangers public health security. Moreover, as single-stranded RNA viruses, antigenic variation and virulence variation can occur during cross-species transmission of both. Last but not least, both can be rapidly rescued by reverse genetic techniques at present [72]. Considering that IAV has the advantages of a relatively systematic theoretical system and has many similarities with SARS-CoV-2, we strongly believe it can be a new benchmark for the most important reference value for SARS-CoV-2 study apart from members of the Coronaviridae family. Moreover, research results of the IAV-host interaction network could provide important references for research ideas about the SARS-CoV-2-host interaction network. The important references include identification of host biomolecules involved in regulating viral replication, and current research trends and future directions of virus-host interaction networks, which could accelerate the study's progress on SARS-CoV-2-host interaction network and lay a theoretical foundation to develop anti-SARS-CoV-2 drugs based on virus-host interaction networks. These important references are summarized as follows.

First and foremost, potential drug targets for SARS-CoV-2 can be rapidly screened in bulk based on these drug targets for IAV, which have been experimentally identified, especially for ncRNAs such as microRNAs, lncRNAs, circRNAs, and vtRNAs. Although the expression difference of some ncRNAs in the host after SARS-CoV-2 infection has been known through high-throughput technology, microRNAs and lncRNAs involved in regulating SARS-CoV-2 replication have not been identified yet [73]. Therefore, we suggest that high-throughput data derived from SARS-CoV-2 infection and previous research results on IAV can be fully combined and quickly validated by interference and overexpression techniques. For example, respectively, as representatives of microRNA and lncRNA, miR-146a and lncRNA-NRAV, which have been determined to regulate IAV replication, can be selected to perform relevant functional validations. Furthermore, the regulatory mechanisms of SARS-CoV-2 replication are worthy of further exploration. For example, whether these biomolecules can directly affect IAV replication by regulating the IAV replication cycle or indirectly affect IAV replication by regulating the innate immune signaling pathways. In addition, based on the research results of IAV, biomolecular regulatory networks associated with SARS-CoV-2 replication can be conducted, which consist of proteins (such as host factors), ncRNAs (such as microRNAs and lncRNAs), sugars, lipids, hormones, and inorganic salts. What's more, research ideas and results about strategies of IAV to antagonize host innate immunity can be used as a reference for further explorations about the new mechanisms of SARS-CoV-2-host interaction. Moreover, IRF3, IRF7, or other knockout mice can be constructed to investigate the molecular mechanisms of innate immune responses against SARS-CoV-2 at the animal level or single-cell level. Besides, new mechanisms of SARS-CoV-2-host interaction at post-translational modification and epigenetic levels can be explored. Additionally, mammal models infected with SARS-CoV-2 can be constructed to simulate cross-species transmission, and the effect of adaptive mutations on viral replication and pathogenicity in the new host can be further investigated by reverse genetics techniques. Finally, when developing SARS-CoV-2 vaccines, lessons should be learned that influenza viruses mutate and lead to vaccine failure. Moreover, ideas and experiences of continuous updating and iteration should be referenced [74].

Recently, co-infection cases of SARS-CoV-2 and IAV have been reported consistently, and a study illustrated that IAV has a unique ability to aggravate SARS-CoV-2 infection [75]. Therefore, when designing anti-SARS-CoV-2 drugs based on biomolecular targets, emphasis should be placed on those host targets with broad-spectrum antiviral activities. For example, the IFITM family was found to significantly inhibit the replication of SARS-CoV-2 and IAV in host cells [76]. Therefore, it is an ideal host target. Moreover, Timothy R Abbott et al. demonstrated a CRISPR-Cas13 system that can effectively identify and degrade the intracellular viral genomes and mRNAs produced during the replication of SARS-CoV-2 and IAV [77]. Given this, the system can be considered as a new antiviral strategy for wider applications in co-infection studies of the two viruses.

## Summary

Infectious diseases have always been a serious threat to social development. In particular, SARS-CoV-2 has been spreading worldwide since 2019 and seriously endangering human health, economic development, and social stability. Currently, there are no targeted drugs for SARS-CoV-2, and the therapeutic schedule is predominantly symptomatic support treatment. Therefore, there is an urgent need to explore antiviral drug targets for SARS-CoV-2 and develop effective clinical drugs. It is imperative to accelerate the study's progress on the SARS-CoV-2-host interaction network to provide a richer range of antiviral drug targets for developing antiviral drugs. Considering that IAV has the advantages of a relatively systematic theoretical system and has many similarities with SARS-CoV-2. We strongly believe the research results of IAV-host interaction network are bound to provide important inspirations for the studies on SARS-CoV-2-host interaction network and accelerate its research progress. To date, only two classes of antiviral drugs are globally approved and available for the treatment of influenza infections. However, they are often associated with limited efficacy and adverse side effects. In addition, the currently available drugs suffer from rapid and extensive emergence of drug resistance. All this highlights the urgent need for developing new antiviral strategies with novel mechanisms of action and with reduced drug resistance potential. Viruses rely on host cellular functions to replicate. Therefore, a thorough understanding of virus-host interaction networks during IAV replication, with a wealth of potential antiviral drug targets, is essential to develop new anti-IAV drugs.

The race between host antiviral innate immunity and viral antagonism of host innate immunity forms virus-host interaction networks, which harbour abundant potential antiviral drug targets. In this review, we systematically summarized the IAV-host interaction networks and SARS-CoV-2-host interaction networks from the perspectives of viral immunology and systems biology to better understand the biological pathogenesis of the disease and proposed new strategies for designing antiviral drugs based on the virus-host interaction networks. In addition, we firmly believe that the mappings of antiviral drug targets focusing on the viral replication cycle or antiviral innate immune signaling pathways could provide richer potential drug targets and a theoretical basis for designing antiviral drugs based on virus-host interaction network. Notably, the virus-host interaction networks harbour three kinds of main antiviral drug targets: viral targets, host targets, and viral antagonism of host innate immunity. To begin with, viral targets are the traditional antiviral drug targets. Theoretically, each of these steps in the viral replication cycle could be used as a potential antiviral drug target. Additionally, these host biomolecules, which regulate viral replication cycle and host innate immune signaling pathways, are selected as a secondary consideration. Last but not least, viral antagonism of host innate immunity in interaction networks could also be used as potential antiviral drug targets, which are the most overlooked in the antiviral drug development process. Unlike the development strategies of antiviral drugs based on virus-host interaction networks proposed by some scholars. Initially, the host targets summarized in this review include non-coding RNAs, sugars, lipids, hormones, and inorganic salts, rather than being limited to proteins. What's more, we systematically summarized the viral antagonism of host innate immunity, which is an often-overlooked antiviral drug target. Finally, we elucidated the molecular mechanisms of IAV-host interaction and SARS-CoV-2-host interaction from the perspectives of viral immunology and systems biology rather than bioinformatics. In conclusion, we summarized a more comprehensive understanding of the virus-host interaction networks from the perspectives of viral immunology and systems biology, providing richer potential drug targets and a theoretical basis for developing antiviral drugs based on the virus-host interaction networks.

## Prospects

Currently, the molecular mechanisms of SARS-CoV-2 cross-species transmission have been poorly investigated. However, the research ideas about cross-species transmission of the avian influenza virus (AIV) are reasonably clear, which could provide important references for SARS-CoV-2 studies. Notably, AIV belongs to the Orthomyxoviridae family, Influenza A virus (IAV) genus. The host barriers that AIV must overcome to initiate a pandemic in humans. Currently, the main approach to simulate cross-species transmission is to construct the mammalian model infected by AIV and acquired adaptive mutations during passaging of AIV. Then the effects of adaptive mutations on the viral receptor-binding capacity of α2,6-SA, viral replication capability, and pathogenicity in a new host are further investigated by reverse genetics techniques [78]. In this way, the key amino acid sites of viral proteins, which regulate cross-species transmission, can be screened, and their molecular mechanisms can be elucidated. It is hoped that these relatively systematic research ideas will accelerate the progress of molecular mechanisms study on SARS-CoV-2 cross-species transmission. Surprisingly, a recent report found that a patient with advanced cancer miraculously healed himself after SARS-CoV-2 infection, and almost all of his tumours disappeared [79]. This clinical discovery seems promising to start a new research journey that is SARS-CoV-2 infection and cancer, which will focus on tumour microenvironment, tumour immunity, inflammation, apoptosis, and autophagy. It will open a new chapter in SARS-CoV-2-host interaction network and provide new ideas for overcoming cancer, which is now a global problem. At present, epidemic (COVID-19) prevention and control in some parts of the world still face enormous challenges of asymptomatic infection. In view of this situation, we suggest the research ideas of Ayuko Hoshino et al. can be referred to explore the host biomarkers in the early stage of SARS-CoV-2 infection by proteomic profiling of extracellular vesicles and particles (EVPs) [80]. In this way, the rapid detection kits for SARS-CoV-2 can be developed for early diagnosis. This research is critical for the early identification and isolation of potential super-spreaders, greatly relieving the global pressure of epidemic prevention and control. At the same time, host biomarkers in COVID-19 patients with various degrees of severity can also be explored based on the research ideas above, to take timely and effective measures for patients with a potentially life-threatening event. There is no doubt that the virus-host interactions are an enduring research topic. After experiencing the joy of overcoming the challenges of infectious disease, we can not help but wonder where the next pandemic virus that threatens human public health might hide. Although IAV already caused four influenza pandemics in 1918, 1957, 1968, and 2009, the next pandemic is still difficult to predict. Furthermore, global warming has directly led to an acceleration of glacial melting in recent years, resulting in the release of ancient viruses. However, we have no way of knowing whether these ancient viruses will pose a serious threat to human health in the future. In addition, we also need time to conclude whether SARS-CoV-2 will coexist with humans for a long time like IAV does. In short, all we can do right now is to constantly extend our understanding of virus-host interactions, accumulate valuable experiences throughout fighting epidemics, always be prepared for long-term struggles against pandemics, and continue to defend human public health with knowledge when facing subsequent pandemic outbreaks.

## Figures and Tables

**Table:** 

SARS-CoV-2	severe acute respiratory syndrome coronavirus 2
IAV	influenza A virus
PRRs	pattern recognition receptors
ISGs	IFN-stimulated genes
ncRNAs	non-coding RNAs
COVID-19	coronavirus disease 2019
ssRNA	single-stranded RNA
dsRNA	double-stranded RNA
vRNPs	viral ribonucleoproteins
vRNAs	viral RNAs
TLRs	Toll-like receptors
RIG-I	retinoic acid-inducible gene-I
MDA5	melanoma differentiation-associated gene 5
MAVS	mitochondrial antiviral signaling
NF-κB	nuclear factor κappa-light-chain-enhancer of activated B cells
IRFs	interferon regulatory factors
TRIMs	tripartite motifs
PACT	PKR activator
TRIF	Toll/interleukin-1 (IL-1) receptor domain-containing adaptor inducing IFN-β
MyD88	myeloid differentiation factor 88
TRAFs	TNF receptor-associated factors
TAK1	TGF-beta-activated kinase 1
IκBα	the alpha inhibitor of NF-κB
TBK1	TANK-binding kinase 1
IKKs	IkappaB kinases
NEMO	NF-κB essential modulator
JAK1	Janus kinase 1
TYK2	tyrosine kinase 2
STATs	signal transducers and activators of transcriptions
ISGF3	IFN-stimulated gene factor 3
ISRE	IFN-stimulated response elements
B4GALNT2	beta-1,4-N-acetyl galactosaminyl transferase 2
SPOCK2	SPARC/osteonectin, cwcv, and kazal-like domains proteoglycan 2
CK2β	casein kinase 2β
FGFR1	fibroblast growth factor receptor 1
NCOA7	nuclear receptor coactivator 7
Nrf2	nuclear factor erythroid 2-related factor 2
IFITM3	interferon-induced transmembrane protein 3
BPIFA1	bactericidal/permeability-increasing fold-containing family member A1
MOV10	Moloney leukaemia virus 10
MxA	myxovirus resistance A
PLSCR1	phospholipid scramblase 1
NCL	Nucleolin
YBX3	Y-box-binding protein 3
eEF1D	eukaryotic translation elongation factor 1 delta
DDXs	DEAD-box proteins
SERTAD3	SERTA domain containing 3
HDACs	histone deacetylases
hnRNP-F	heterogeneous nuclear ribonucleoprotein F
PKP2	plakophilin 2
RTF2	replication termination factor 2
Cyps	Cyclophilins
RBL2	RuvB-like protein 2
SOD1	superoxide dismutase1
Mx1	myxovirus resistance 1
hnRNP A2/B1	heterogeneous nuclear ribonucleoprotein A2/B1
Nups	Nucleoporins
Rae1	ribonucleic acid export 1
ZAPS	zinc-finger antiviral protein shorter isoform
ZFP36L1	zinc finger protein 36, C3H type-like 1
HDM2	human homologue of mouse double minute 2
Grail	gene related to anergy in lymphocytes
USPs	ubiquitin-specific proteases
ZAPL	zinc-finger antiviral protein longer isoform
ARHGAP21	Rho GTPase activating protein 21
AnxA6	annexin A6
Viperin	virus inhibitory protein, endoplasmic reticulum-associated, interferon-inducible
AGOs	Argonautes
Dot1L	disruptor of telomeric silencing 1-like
eIF4B	eukaryotic translation initiation factor 4B
FBXW7	F-box and WD repeat domain containing 7
FGF2	fibroblast growth factor 2
FKBP5	FK506 binding protein 5
GBP5	guanylate binding protein 5
HERP	homocysteine-induced ER protein
MCCC1	methylcrotonoyl-CoA carboxylase 1
MVP	major vault protein
NF90	nuclear factor 90
NLRC5	NLR family CARD domain containing 5
NMB	neuromedin B
RSK2	ribosomal S6 kinase 2
S1PR1	sphingosine-1-phosphate receptor 1
SLFN14	Schlafen 14
SNW1	SNW domain-containing protein 1
SPL	sphingosine 1-phosphate lyase
TIP60	Tat-interactive protein 60 kDa
ZMPSTE24	zinc metallopeptidase STE24
EGFR	epidermal growth factor receptor
FAK	focal adhesion kinase
FFAR2	free fatty acid receptor 2
GBA	glucosylceramidase
LY6E	lymphocyte antigen 6E
MCOLN2	mucolipin-2
PLC-γ1	phosphoinositide-specific phospholipase C γ1
PLD2	phospholipase D2
RNASEK	ribonuclease kappa
SLC35A1	solute carrier family 35 member A1
ACP2	acid phosphatase 2
CtsW	cathepsin W
EPS8	epidermal growth factor receptor pathway substrate 8
GRK2	G protein-coupled receptor kinase 2
PEPD	Prolidase
AP2M1	AP-2 complex subunit mu
Hsps	heat shock proteins
SH3GLB1	SH3-domain GRB2-like endophilin B2
IREF-2	influenza virus replication factor-2
JNK	c-Jun N-terminal kinase
PRPF8	pre-mRNA processing factor 8
CDC25B	cell division cycle 25 B
CHD1	chromodomain helicase DNA binding protein 1
CLE	C14orf166
CTR1	copper transporter 1
DR1	down-regulator of transcription 1
eIF4A3	eukaryotic translation initiation factor 4A3
GPS1	G protein pathway suppressor 1
HMGB1	high-mobility group box 1
PKC	protein kinase C
PPP6C	protein phosphatase 6 catalytic subunit
FMRP	Fragile X mental retardation protein
LYAR	Ly-1 antibody reactive clone
CHD3	chromodomain helicase DNA binding protein 3
Hsc70	heat shock cognate protein 70
KIF18A	kinesin family member 18A
MLC	myosin light chain
NOX2	NADPH oxidase 2
PRC2	polycomb repressive complex 2
RanBP3	Ran-binding protein 3
SGK1	serum-glucocorticoid regulated kinase 1
SK	sphingosine kinase
XPO1	exportin 1
CDKs	cyclin-dependent kinases
ERI1	exoribonuclease 1
RRP1B	ribosomal RNA processing 1 homologue B
ZC3H11A	zinc finger CCCH-type containing 11A
eEF1G	eukaryotic elongation factor 1 gamma
AIMP2	aminoacyl-tRNA synthetase-interacting multifunctional protein 2
CNOT4	ccr4-Not transcription complex subunit 4
CRL4	the CUL4-RING E3 ubiquitin ligase
Cdc42	cell division cycle 42
NUMA1	nuclear mitotic apparatus protein 1
UBR4	ubiquitin protein ligase E3 component N-recognin 4
F1β	F1Fo-ATPase
RACK1	receptor for activated C kinase 1
CUEDC2	CUE domain containing 2
DPF2	double plant homeodomain fingers 2
EZH2	enhancer of zeste homologue 2
FADD	Fas-associated death domain
FAT10	human leukocyte antigen F-associated transcript 10
GLDC	glycine decarboxylase
IFI44L	IFN-induced protein 44-like
KHSRP	K-homology splicing regulatory protein
MKs	MAP kinase-activated protein kinases
MKP5	MAPK phosphatase 5
NDRG1	N-myc downstream-regulated gene 1
NEDD4	neuronal precursor cell expressed developmentally downregulated 4
PARP1	poly(ADP-ribose) polymerase 1
PGRN	Progranulin
Rubicon	RUN domain Beclin-1-interacting cysteine-rich-containing
SOCSs	suppressors of cytokine signaling
TAP1	antigen peptide transporter 1
ZBTB25	zinc finger and BTB domain containing 25
NIK	NF-κΒ inducing kinase
NFKBIB	NF-κB inhibitor beta
ATP6V1A	ATPase catalytic subunit A
GALNT3	GalNAc transferase 3
USP3	ubiquitin-specific protease 3
ARCN1	archain 1
NS1ABP	non-structural-1A binding protein
PLK4	polo-like kinase 4
VIM	Vimentin
OAS2	2'-5'-oligoadenylate synthase 2
COX6C	cytochrome c oxidase subunit 6C
LIF	leukaemia inhibitory factor
MCPIP1	monocyte chemoattractant protein-induced protein 1
PTP1B	protein tyrosine phosphatase 1B
PKR	protein kinase R
SM	Sphingomyelin
PD1	protectin D1
CORT	Corticosterone
FLNA	filamin A
MDM2	murine double minute 2
HCV	hepatitis C virus
EV	Ebola virus
HIV	human immunodeficiency virus
DV	dengue virus
ASFV	African swine fever virus
IBV	influenza B virus
ACE2	angiotensin-converting enzyme 2
TMPRSS2	transmembrane protease, serine 2
HMGB1	high mobility group protein B1
mTOR	mammalian target of rapamycin
PI3K	phosphatidylinositol-3-kinase
Raf	rapidly accelerated fibrosarcoma
SRPKs	SRSF protein kinases
VPS34	vacuolar protein sorting 34
CH25H	cholesterol 25-hydroxylase
LARP1	La-related protein 1
ZAP	zinc-finger antiviral protein
AP-1	activator protein 1
CK2	casein kinase II
p38 MAPK	p38 mitogen-activated protein kinase
MERS-CoV	Middle East respiratory syndrome coronavirus

## References

[1] Meineke R, Rimmelzwaan G, Elbahesh H. Influenza virus infections and cellular kinases. Viruses. 2019;11:2. 10.3390/v11020171 PMC6410056 30791550

[2] Chen X, Liu S, Goraya MU, et al. Host immune response to influenza A virus infection. Front Immunol. 2018;9:320. 29556226 10.3389/fimmu.2018.00320 PMC5845129

[3] Engelhardt OG, Fodor E. Functional association between viral and cellular transcription during influenza virus infection. Rev Med Virol. 2006;16(5):329–345. 16933365 10.1002/rmv.512

[4] Yewdell JW, Ince WL. Virology. Frameshifting to PA-X influenza. Science. 2012;337(6091):164–165. 22798590 10.1126/science.1225539 PMC3777247

[5] Pauli EK, Schmolke M, Wolff T, et al. Influenza A virus inhibits type I IFN signaling via NF-kappaB-dependent induction of SOCS-3 expression. PLoS Pathog. 2008;4(11):e1000196. 18989459 10.1371/journal.ppat.1000196 PMC2572141

[6] Zhang F, Sun X, Zhu Y, et al. Downregulation of miR-146a inhibits influenza A virus replication by enhancing the type I interferon response in vitro and in vivo. Biomed Pharmacother. 2019;111:740–750. 30611999 10.1016/j.biopha.2018.12.103

[7] Ouyang J, Zhu X, Chen Y, et al. NRAV, a long noncoding RNA, modulates antiviral responses through suppression of interferon-stimulated gene transcription. Cell Host Microbe. 2014;16(5):616–626. 25525793 10.1016/j.chom.2014.10.001 PMC7104942

[8] Yu T, Ding Y, Zhang Y, et al. Circular RNA GATAD2A promotes H1N1 replication through inhibiting autophagy. Vet Microbiol. 2019;231:238–245. 30955816 10.1016/j.vetmic.2019.03.012

[9] Li F, Chen Y, Zhang Z, et al. Robust expression of vault RNAs induced by influenza A virus plays a critical role in suppression of PKR-mediated innate immunity. Nucleic Acids Res. 2015;43(21):10321–10337. 26490959 10.1093/nar/gkv1078 PMC4666359

[10] Liu S, Wang T, Wang M, et al. Epigenetic modification Is regulated by the interaction of influenza A virus nonstructural protein 1 with the De Novo DNA methyltransferase DNMT3B and subsequent transport to the cytoplasm for K48-linked polyubiquitination. J Virol. 2019;93:7. 10.1128/JVI.01587-18 PMC6430541 30651365

[11] Adamson CS, Chibale K, Goss RJM, et al. Antiviral drug discovery: preparing for the next pandemic. Chem Soc Rev. 2021;50(6):3647–3655. 33524090 10.1039/d0cs01118e

[12] Meganck RM, Baric RS. Developing therapeutic approaches for twenty-first-century emerging infectious viral diseases. Nat Med. 2021;27(3):401–410. 33723456 10.1038/s41591-021-01282-0

[13] de Chassey B, Meyniel-Schicklin L, Vonderscher J, et al. Virus-host interactomics: new insights and opportunities for antiviral drug discovery. Genome Med. 2014;6(11):115. 25593595 10.1186/s13073-014-0115-1 PMC4295275

[14] Loregian A, Mercorelli B, Nannetti G, et al. Antiviral strategies against influenza virus: towards new therapeutic approaches. Cell Mol Life Sci. 2014;71(19):3659–3683. 24699705 10.1007/s00018-014-1615-2 PMC11114059

[15] Pizzorno A, Padey B, Terrier O, et al. Drug repurposing approaches for the treatment of influenza viral infection: reviving old drugs to fight against a long-lived enemy. Front Immunol. 2019;10:531. 30941148 10.3389/fimmu.2019.00531 PMC6434107

[16] Watanabe T, Kawaoka Y. Influenza virus-host interactomes as a basis for antiviral drug development. Curr Opin Virol. 2015;14:71–78. 26364134 10.1016/j.coviro.2015.08.008 PMC5380926

[17] Watanabe T, Kawakami E, Shoemaker J, et al. Influenza virus-host interactome screen as a platform for antiviral drug development. Cell Host Microbe. 2014;16(6):795–805. 25464832 10.1016/j.chom.2014.11.002 PMC4451456

[18] Shaw ML. The host interactome of influenza virus presents new potential targets for antiviral drugs. Rev Med Virol. 2011;21(6):358–369. 21823192 10.1002/rmv.703 PMC3207218

[19] Zhou YW, Xie Y, Tang L-S, et al. Therapeutic targets and interventional strategies in COVID-19: mechanisms and clinical studies. Signal Transduct Target Ther. 2021;6(1):317. 34446699 10.1038/s41392-021-00733-x PMC8390046

[20] Artese A, Svicher V, Costa G, et al. Current status of antivirals and druggable targets of SARS CoV-2 and other human pathogenic coronaviruses. Drug Resist Updat. 2020;53:100721. 33132205 10.1016/j.drup.2020.100721 PMC7448791

[21] Liu X, Huuskonen S, Laitinen T, et al. SARS-CoV-2-host proteome interactions for antiviral drug discovery. Mol Syst Biol. 2021;17(11):e10396. 34709727 10.15252/msb.202110396 PMC8552907

[22] Te Velthuis AJ, Fodor E. Influenza virus RNA polymerase: insights into the mechanisms of viral RNA synthesis. Nat Rev Microbiol. 2016;14(8):479–493. 27396566 10.1038/nrmicro.2016.87 PMC4966622

[23] Reikine S, Nguyen JB, Modis Y, et al. Pattern recognition and signaling mechanisms of RIG-I and MDA5. Front Immunol. 2014;5:342. 25101084 10.3389/fimmu.2014.00342 PMC4107945

[24] Gack MU, Albrecht RA, Urano T, et al. Influenza A virus NS1 targets the ubiquitin ligase TRIM25 to evade recognition by the host viral RNA sensor RIG-I. Cell Host Microbe. 2009;5(5):439–449. 19454348 10.1016/j.chom.2009.04.006 PMC2737813

[25] Rajsbaum R, Albrecht RA, Wang MK, et al. Species-specific inhibition of RIG-I ubiquitination and IFN induction by the influenza A virus NS1 protein. PLoS Pathog. 2012;8(11):e1003059. 23209422 10.1371/journal.ppat.1003059 PMC3510253

[26] Tawaratsumida K, Phan V, Hrincius ER, et al. Quantitative proteomic analysis of the influenza A virus nonstructural proteins NS1 and NS2 during natural cell infection identifies PACT as an NS1 target protein and antiviral host factor. J Virol. 2014;88(16):9038–9048. 24899174 10.1128/JVI.00830-14 PMC4136281

[27] Yan N, Chen ZJ. Intrinsic antiviral immunity. Nat Immunol. 2012;13(3):214–222. 22344284 10.1038/ni.2229 PMC3549670

[28] Du Y, Yang F, Wang Q, et al. Influenza a virus antagonizes type I and type II interferon responses via SOCS1-dependent ubiquitination and degradation of JAK1. Virol J. 2020;17(1):74. 32532301 10.1186/s12985-020-01348-4 PMC7291424

[29] Khongnomnan K, Makkoch J, Poomipak W, et al. Human miR-3145 inhibits influenza A viruses replication by targeting and silencing viral PB1 gene. Exp Biol Med (Maywood. 2015;240(12):1630–1639. 26080461 10.1177/1535370215589051 PMC4935342

[30] Song L, Liu H, Gao S, et al. Cellular microRNAs inhibit replication of the H1N1 influenza A virus in infected cells. J Virol. 2010;84(17):8849–8860. 20554777 10.1128/JVI.00456-10 PMC2919005

[31] Kumar A, Kumar A, Ingle H, et al. MicroRNA hsa-miR-324-5p Suppresses H5N1 virus replication by targeting the viral PB1 and host CUEDC2. J Virol. 2018;92:19. 10.1128/JVI.01057-18 PMC6146810 30045983

[32] Ingle H, Kumar S, Raut AA, et al. The microRNA miR-485 targets host and influenza virus transcripts to regulate antiviral immunity and restrict viral replication. Sci Signal. 2015;8(406):ra126. 26645583 10.1126/scisignal.aab3183

[33] Wang R, Zhang Y-Y, Lu J-S, et al. The highly pathogenic H5N1 influenza A virus down-regulated several cellular MicroRNAs which target viral genome. J Cell Mol Med. 2017;21(11):3076–3086. 28609011 10.1111/jcmm.13219 PMC5661113

[34] Cui H, Zhang C, Zhao Z, et al. Identification of cellular microRNA miR-188-3p with broad-spectrum anti-influenza A virus activity. Virol J. 2020;17(1):12. 32000791 10.1186/s12985-020-1283-9 PMC6993346

[35] Bavagnoli L, Campanini G, Forte M, et al. Identification of a novel antiviral micro-RNA targeting the NS1 protein of the H1N1 pandemic human influenza virus and a corresponding viral escape mutation. Antiviral Res. 2019;171:104593. 31470040 10.1016/j.antiviral.2019.104593

[36] Ma YJ, Yang J, Fan X-L, et al. Cellular microRNA let-7c inhibits M1 protein expression of the H1N1 influenza A virus in infected human lung epithelial cells. J Cell Mol Med. 2012;16(10):2539–2546. 22452878 10.1111/j.1582-4934.2012.01572.x PMC3823446

[37] Zhao L, Zhu J, Zhou H, et al. Identification of cellular microRNA-136 as a dual regulator of RIG-I-mediated innate immunity that antagonizes H5N1 IAV replication in A549 cells. Sci Rep. 2015;5:14991. 26450567 10.1038/srep14991 PMC4598873

[38] Zhao L, Zhang X, Wu Z, et al. The downregulation of MicroRNA hsa-miR-340-5p in IAV-infected A549 cells suppresses viral replication by targeting RIG-I and OAS2. Mol Ther Nucleic Acids. 2019;14:509–519. 30753994 10.1016/j.omtn.2018.12.014 PMC6370596

[39] Hsu AC, Dua K, Starkey MR, et al. MicroRNA-125a and -b inhibit A20 and MAVS to promote inflammation and impair antiviral response in COPD. JCI Insight. 2017;2(7):e90443. 28405612 10.1172/jci.insight.90443 PMC5374076

[40] Rosenberger CM, Podyminogin RL, Diercks AH, et al. miR-144 attenuates the host response to influenza virus by targeting the TRAF6-IRF7 signaling axis. PLoS Pathog. 2017;13(4):e1006305. 28380049 10.1371/journal.ppat.1006305 PMC5393898

[41] Wang K, Lai C, Gu H, et al. miR-194 inhibits innate antiviral immunity by targeting FGF2 in influenza H1N1 virus infection. Front Microbiol. 2017;8:2187. 29163456 10.3389/fmicb.2017.02187 PMC5674008

[42] Guo M, Li F, Ji J, et al. Inhibition of miR-93 promotes interferon effector signaling to suppress influenza A infection by upregulating JAK1. Int Immunopharmacol. 2020;86:106754. 32652502 10.1016/j.intimp.2020.106754

[43] Maarouf M, Chen B, Chen Y, et al. Identification of lncRNA-155 encoded by MIR155HG as a novel regulator of innate immunity against influenza A virus infection. Cell Microbiol. 2019;21(8):e13036. 31045320 10.1111/cmi.13036

[44] Chai W, Li J, Shangguan Q, et al. Lnc-ISG20 inhibits influenza A virus replication by enhancing ISG20 expression. J Virol. 2018;92:16. 10.1128/JVI.00539-18 PMC6069214 29899085

[45] Li X, Guo G, Lu M, et al. Long noncoding RNA Lnc-MxA inhibits beta interferon transcription by forming RNA-DNA triplexes at its promoter. J Virol. 2019;93:21. 10.1128/JVI.00786-19 PMC6803265 31434735

[46] Wang Q, Zhang D, Feng W, et al. Long noncoding RNA TSPOAP1 antisense RNA 1 negatively modulates type I IFN signaling to facilitate influenza A virus replication. J Med Virol. 2022;94(2):557–566. 30968963 10.1002/jmv.25483

[47] Zhao L, Xia M, Wang K, et al. A long Non-coding RNA IVRPIE promotes host antiviral immune responses through regulating interferon beta1 and ISG expression. Front Microbiol. 2020;11:260. 32153544 10.3389/fmicb.2020.00260 PMC7044153

[48] Wang J, Zhang Y, Li Q, et al. Influenza virus exploits an interferon-independent lncRNA to preserve viral RNA synthesis through stabilizing viral RNA polymerase PB1. Cell Rep. 2019;27(11):3295–3304. e4. 31189112 10.1016/j.celrep.2019.05.036

[49] Wang J, Wang Y, Zhou R, et al. Host long noncoding RNA lncRNA-PAAN regulates the replication of influenza A virus. Viruses. 2018;10:6. 10.3390/v10060330 PMC6024364 29914164

[50] Wang P, Xu J, Cao X, et al. An interferon-independent lncRNA promotes viral replication by modulating cellular metabolism. Science. 2017;358(6366):1051–1055. 29074580 10.1126/science.aao0409

[51] Qu Z, Meng F, Shi J, et al. A novel intronic Circular RNA antagonizes influenza virus by absorbing a microRNA that degrades CREBBP and accelerating IFN-beta production. mBio. 2021;12(4):e0101721. 34281396 10.1128/mBio.01017-21 PMC8406138

[52] Kohio HP, Adamson AL. Glycolytic control of vacuolar-type ATPase activity: a mechanism to regulate influenza viral infection. Virology. 2013;444(1-2):301–309. 23876457 10.1016/j.virol.2013.06.026

[53] Morita M, Kuba K, Ichikawa A, et al. The lipid mediator protectin D1 inhibits influenza virus replication and improves severe influenza. Cell. 2013;153(1):112–125. 23477864 10.1016/j.cell.2013.02.027

[54] Peretz J, Pekosz A, Lane AP, et al. Estrogenic compounds reduce influenza A virus replication in primary human nasal epithelial cells derived from female, but not male, donors. Am J Physiol Lung Cell Mol Physiol. 2016;310(5):L415–L425. 26684252 10.1152/ajplung.00398.2015 PMC4773846

[55] Read SA, O’Connor KS, Suppiah V, et al. Zinc is a potent and specific inhibitor of IFN-lambda3 signalling. Nat Commun. 2017;8:15245. 28513591 10.1038/ncomms15245 PMC5442324

[56] Luo Z, Liu L-F, Jiang Y-N, et al. Novel insights into stress-induced susceptibility to influenza: corticosterone impacts interferon-beta responses by Mfn2-mediated ubiquitin degradation of MAVS. Signal Transduct Target Ther. 2020;5(1):202. 32943610 10.1038/s41392-020-00238-z PMC7499204

[57] Engel DA. The influenza virus NS1 protein as a therapeutic target. Antiviral Res. 2013;99(3):409–416. 23796981 10.1016/j.antiviral.2013.06.005 PMC4373342

[58] Assil S, Webster B, Dreux M, et al. Regulation of the host antiviral state by intercellular communications. Viruses. 2015;7(8):4707–4733. 26295405 10.3390/v7082840 PMC4576201

[59] Yang C, Liu X, Cheng T, et al. LYAR suppresses beta interferon induction by targeting phosphorylated interferon regulatory factor 3. J Virol. 2019;93(21):e00769-19. 10.1128/JVI.00769-19 PMC6803289 31413131

[60] Schaefer MH, Lopes TJS, Mah N, et al. Adding protein context to the human protein-protein interaction network to reveal meaningful interactions. PLoS Comput Biol. 2013;9(1):e1002860. 23300433 10.1371/journal.pcbi.1002860 PMC3536619

[61] Steuerman Y, Cohen M, Peshes-Yaloz N, et al. Dissection of influenza infection In vivo by single-cell RNA sequencing. Cell Syst. 2018;6(6):679–691. e4. 29886109 10.1016/j.cels.2018.05.008 PMC7185763

[62] Ramos I, Smith G, Ruf-Zamojski F, et al. Innate immune response to influenza virus at single-cell resolution in human epithelial cells revealed paracrine induction of interferon lambda 1. J Virol. 2019;93:20. 10.1128/JVI.00559-19 PMC6798124 31375585

[63] Zhou W, Cheng X, Zhang Y, et al. Effect of Liuwei Dihuang decoction, a traditional Chinese medicinal prescription, on the neuroendocrine immunomodulation network. Pharmacol Ther. 2016;162:170–178. 26896567 10.1016/j.pharmthera.2016.02.004

[64] Gordon DE, Jang GM, Bouhaddou M, et al. A SARS-CoV-2 protein interaction map reveals targets for drug repurposing. Nature. 2020;583(7816):459–468. 32353859 10.1038/s41586-020-2286-9 PMC7431030

[65] Hillen HS, Kokic G, Farnung L, et al. Structure of replicating SARS-CoV-2 polymerase. Nature. 2020;584(7819):154–156. 32438371 10.1038/s41586-020-2368-8

[66] Hoffmann M, Kleine-Weber H, Schroeder S, et al. SARS-CoV-2 cell entry depends on ACE2 and TMPRSS2 and Is blocked by a clinically proven protease inhibitor. Cell. 2020;181(2):271–280. e8. 32142651 10.1016/j.cell.2020.02.052 PMC7102627

[67] Bojkova D, Klann K, Koch B, et al. Proteomics of SARS-CoV-2-infected host cells reveals therapy targets. Nature. 2020;583(7816):469–472. 32408336 10.1038/s41586-020-2332-7 PMC7616921

[68] Bouhaddou M, Memon D, Meyer B, et al. The global phosphorylation landscape of SARS-CoV-2 infection. Cell. 2020;182(3):685–712. e19. 32645325 10.1016/j.cell.2020.06.034 PMC7321036

[69] Klann K, Bojkova D, Tascher G, et al. Growth factor receptor signaling inhibition prevents SARS-CoV-2 replication. Mol Cell. 2020;80(1):164–174. e4. 32877642 10.1016/j.molcel.2020.08.006 PMC7418786

[70] El Baba R, Herbein G. Management of epigenomic networks entailed in coronavirus infections and COVID-19. Clin Epigenetics. 2020;12(1):118. 32758273 10.1186/s13148-020-00912-7 PMC7404079

[71] Yang H, Lyu Y, Hou F, et al. SARS-CoV-2 infection and the antiviral innate immune response. J Mol Cell Biol. 2020;12(12):963–967. 33377937 10.1093/jmcb/mjaa071 PMC7798998

[72] Thi Nhu Thao T, Labroussaa F, Ebert N, et al. Rapid reconstruction of SARS-CoV-2 using a synthetic genomics platform. Nature. 2020;582(7813):561–565. 32365353 10.1038/s41586-020-2294-9

[73] Kim WR, Park EG, Kang K-W, et al. Expression analyses of MicroRNAs in hamster lung tissues infected by SARS-CoV-2. Mol Cells. 2020;43(11):953–963. 33199671 10.14348/molcells.2020.0177 PMC7700842

[74] Korber B, Fischer WM, Gnanakaran S, et al. Tracking changes in SARS-CoV-2 spike: evidence that D614G increases infectivity of the COVID-19 virus. Cell. 2020;182(4):812–827. e19. 32697968 10.1016/j.cell.2020.06.043 PMC7332439

[75] Bai L, Zhao Y, Dong J, et al. Coinfection with influenza A virus enhances SARS-CoV-2 infectivity. Cell Res. 2021;31(4):395–403. 33603116 10.1038/s41422-021-00473-1 PMC7890106

[76] Shi G, Kenney AD, Kudryashova E, et al. Opposing activities of IFITM proteins in SARS-CoV-2 infection. EMBO J. 2021;40(3):e106501. 33270927 10.15252/embj.2020106501 PMC7744865

[77] Abbott TR, Dhamdhere G, Liu Y, et al. Development of CRISPR as an antiviral strategy to Combat SARS-CoV-2 and influenza. Cell. 2020;181(4):865–876. e12. 32353252 10.1016/j.cell.2020.04.020 PMC7189862

[78] Ping J, Dankar SK, Forbes NE, et al. PB2 and hemagglutinin mutations are major determinants of host range and virulence in mouse-adapted influenza A virus. J Virol. 2010;84(20):10606–10618. 20702632 10.1128/JVI.01187-10 PMC2950562

[79] Challenor S, Tucker D. SARS-CoV-2-induced remission of Hodgkin lymphoma. Br J Haematol. 2021;192(3):415. 33386647 10.1111/bjh.17116

[80] Hoshino A, Kim HS, Bojmar L, et al. Extracellular vesicle and particle biomarkers define multiple human cancers. Cell. 2020;182(4):1044–1061. e18. 32795414 10.1016/j.cell.2020.07.009 PMC7522766

